# Picornavirus Cellular Remodeling: Doubling Down in Response to Viral-Induced Inflammation

**DOI:** 10.1007/s40588-020-00138-4

**Published:** 2020-04-17

**Authors:** Alexis Bouin, Bert L. Semler

**Affiliations:** 1 Department of Microbiology & Molecular Genetics and Center for Virus Research, School of Medicine, University of California, Med Sci Bldg, Room B237, Irvine, CA 92697-4025, USA

**Keywords:** Picornavirus, Rhinovirus, Coxsackievirus, Inflammation, Remodeling, Asthma, Cystic fibrosis, Myocarditis

## Abstract

**Purpose of Review:**

To highlight recent findings on how picornavirus infections of the airways and cardiac tissues impact cellular inflammation and remodeling events.

**Recent Findings:**

Recent published work has revealed that although many picornavirus infections appear to be initially asymptomatic, there are significant disease sequelae that result from chronic or persistent infections and the long-term, pathogenic effects on host tissues.

**Summary:**

Because many acute picornavirus infections are asymptomatic, it is difficult to diagnose these pathologies at the early stages of disease. As a result, we must rely on preventative measures (i.e., vaccination) or discover novel treatments to reverse tissue damage and remodeling in affected individuals. Both of these strategies will require a comprehensive knowledge of virus-and cell-specific replication determinants and how these processes induce pathogenic effects in infected cells and tissues.

## Introduction

Picornaviruses are small non-enveloped viruses whose sizes vary from 30 to 32 nm. The capsid is composed of 60 identical protomers assembled in an icosahedral structure protecting the viral genome, which consists of a non-segmented, positive-strand RNA ranging from 6.7 to 10.1 kb. The family *Picornaviridae* belongs to the order *Picornavirales*. To date, more than 110 species have been described, grouped in more than 47 genera [1, 2]. These viruses are primarily transmitted by the fecal-oral route or via saliva and respiratory droplets. They can infect a number of different organs, including the central nervous system, heart, liver, skin, gastrointestinal tract, and upper respiratory tract. Each species uses a dedicated receptor(s) to enter the host cell, and viral replication steps take place in the cytoplasm.

Following infection of host cells, the innate immune system detects viral components and triggers a response. It is known that melanoma differentiation–associated gene 5 (MDA5) recognizes dsRNA of picornaviruses [3, 4]. Moreover, retinoic acid–inducible gene I (RIG-I) is cleaved during picornavirus infection, suggesting a role in the innate response to these viruses [5]. MDA5 and RIG-I both stimulate the production of cytokines, which allows the recruitment of immune cells that will trigger a response to eliminate the pathogenic agent. These inflammatory mechanisms are dependent on host cells (and their tissue of origin) as well as the virus [6]. In most cases, such inflammatory mechanisms are good for the host; however, on some occasions an excessive response is triggered and is detrimental to the host by inducing tissue remodeling, resulting in disease. In this review, we will summarize the most recent findings on the inflammation and remodeling events after picornavirus infections of the respiratory airways and cardiac tissue.

## Membrane Remodeling

It is well known that picornaviruses divert cellular membranes of infected cells to create replication organelles. This topic has been reviewed recently in [7]. These replication organelles are composed of hijacked intracellular membranes, re-purposed by the viral 3A(B) protein to enhance picornavirus replication [8]. It is thought that they serve a dual purpose of replication complex formation and compartmentalization of the viral RNA [9], allowing the virus to escape from RNA and pathogen intracellular sensing, and subsequently, the immune response of the host. Additionally, members of the enterovirus genus of the picornavirus family have been reported to re-purpose autophagosomes and use them to release progeny virions from the cell in an alternative, non-lytic manner, allowing the viruses to be secreted without killing host cells and to display new properties in dissemination [10]. These mechanisms have been reviewed recently [11, 12].

## Airway Remodeling

Respiratory infections by picornaviruses are a common phenomenon, ranging from the common cold induced by human rhinovirus (HRV) to more life-threatening conditions induced by the expanding outbreaks of enterovirus D68 (EV-D68) infections. Here, we will discuss recent findings on the role of picornavirus infections in asthma and cystic fibrosis and the exacerbation of these conditions.

### Asthma

Asthma is a chronic respiratory disease characterized by wheezing, shortness of breath, chest tightness, cough, and variable airflow limitations. These symptoms are not continuous and can be caused by a number of factors such as exercise, allergens, irritant exposure, or viral respiratory infections [13]. An ever-increasing number of studies document the involvement of HRV in asthma [14].

**Fig. 1:**
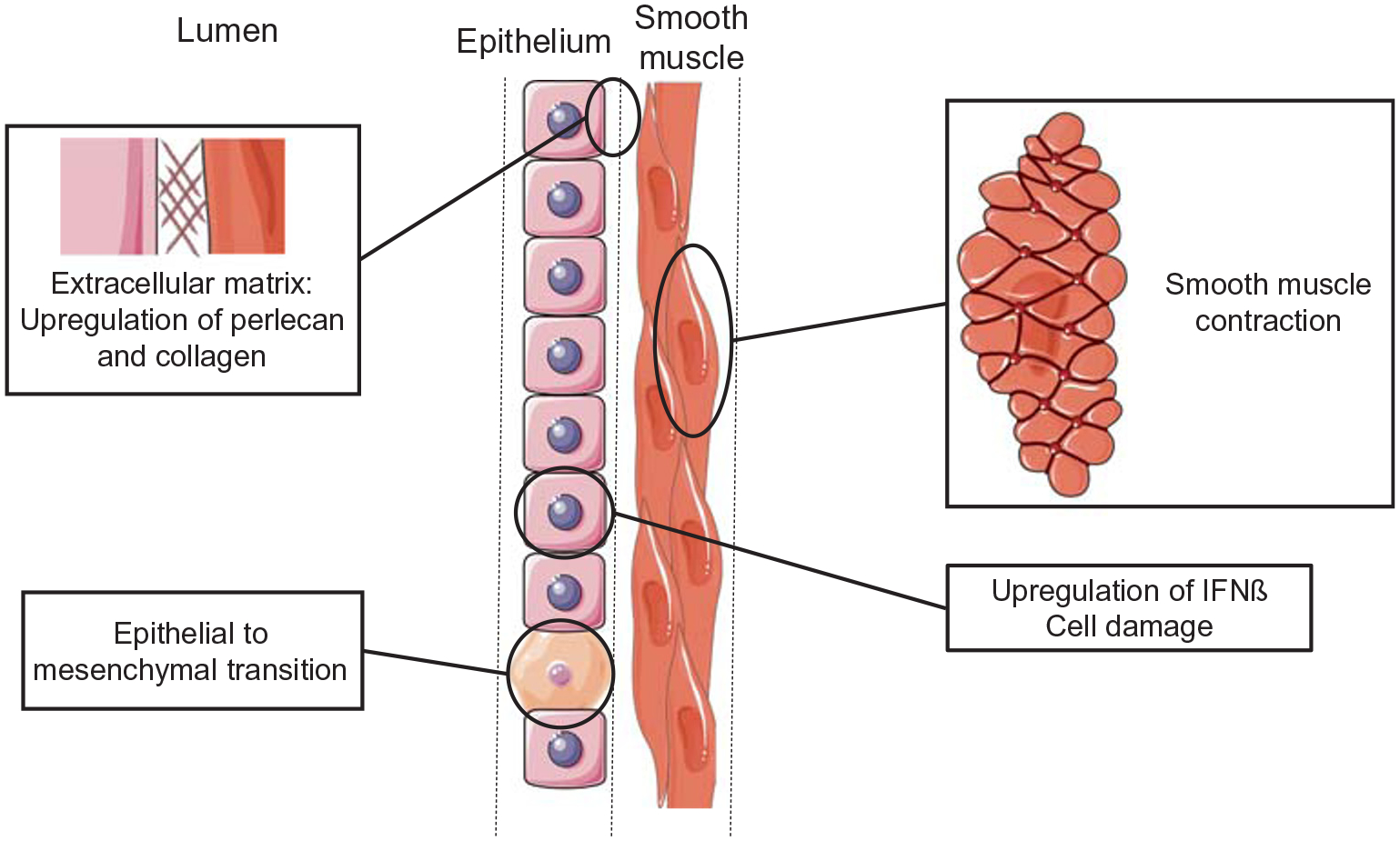
Remodeling of respiratory airway by human rhinoviruses. Following infection and replication, respiratory tissues can undergo changes affecting both cellular morphology and biology as well as the extracellular compartments. The text boxes illustrate the respiratory tissue components that are affected by rhinovirus infections

Airway remodeling is an important hallmark of asthma, and deposition of increased levels of extracellular matrix (ECM) protein is one of the events leading to that remodeling. HRVs are able to increase deposition of perlecan, an extracellular matrix (ECM) protein, collagen V, and matrix-bound vascular endothelial growth factor in human bronchial epithelial cells [15]. Moreover, it has been reported that HRVs are able to induce the process of epithelial-mesenchymal transition in bronchial cell lines, and are even more efficient when synergizing with TGF-β1, potentially inducing an increased ECM protein deposition [16•]. ECM is known to regulate smooth muscle contraction [17]; the remodeling of this component decreases airway flow and could, in part, explain the role of HRV in asthma. Moreover, infection of monocytes by HRV upregulates ORMDL3 (a sphingolipid biosynthesis regulator), leading to increased levels of IFN-β and the endoplasmic reticulum chaperone BiP (HSPA5). This effect was enhanced in cells harboring genes associated with asthma [18]. Interestingly, it has been shown that IL-6, IL-8, and RANTES levels are lower and TGF-β1 levels are higher when cells are infected by HRV in an atopic asthmatic environment compared with a healthy environment. These results show that in an asthmatic patient, inflammation is decreased, resulting in higher levels of viral replication and increased cell damage [19], providing a plausible explanation for the loss of function of airway cells. A plausible scheme for cellular and tissue remodeling induced during human rhinovirus replication is shown in Fig. 1.

### Cystic Fibrosis

Cystic fibrosis is an autosomal disorder caused by a mutation in the cystic fibrosis transmembrane conductance regulator protein. This mutation induces a change in the activity of chloride and sodium channels of sweat- and mucus-producing cells, resulting in thick and sticky mucus, setting an optimal environment for pathogen replication, especially pseudomonas [20]. It has been reported that HRVs are the most common viruses found in patients suffering from cystic fibrosis [21, 22], present in up to 43% of children under the age of five suffering from cystic fibrosis [23]. However, the precise role of HRV in cystic fibrosis requires further investigation, as it is unclear if chronic infections by these viruses increase the disease sequelae by inducing inflammatory responses or are just a secondary outcome [24].

HRV infection results in the upregulation of the chemokines CXCL10, CXCL11, and CXCL9 in patients suffering from cystic fibrosis, recruiting monocytes to the site of infection [25]. A recent study showed that different groups of rhinoviruses (differentiated by receptor usage) induced different effects on primary isolates of bronchial epithelial cells (BEC) from patients with cystic fibrosis. The so-called major HRV group infection of cystic fibrosis BEC yielded decreased interferon (IFN) responses compared with control BECs. In contrast, minor HRV group infections induced increased levels of IFN as well as increased expression of pattern recognition receptors that act as pathogen sensors [26]. Nevertheless, the association between HRVand cystic fibrosis requires more comprehensive studies since children suffering from this disease appear to be more susceptible to infection and for longer periods of time [27]. It is clear that HRVs are able to profoundly change cellular biology. For example, an in vitro study on alveolar epithelial cell monolayers infected by HRV analyzed differentially expressed genes. During the early steps of infection (up to 6 h post-infection), an upregulation of genes involved in the inflammatory response was observed. Subsequently, these viruses upregulate genes responsible for apoptosis, anti-apoptosis, blood vessel morphogenesis, and wound healing (12 h post-infection). Finally at late times of infection (24 h and 48 h post-infection), a downregulation of genes involved in airway remodeling events is observed [28]. Altogether, these results highlight a potential role for HRV in exacerbating the symptoms of cystic fibrosis.

## Myocarditis

**Fig. 2:**
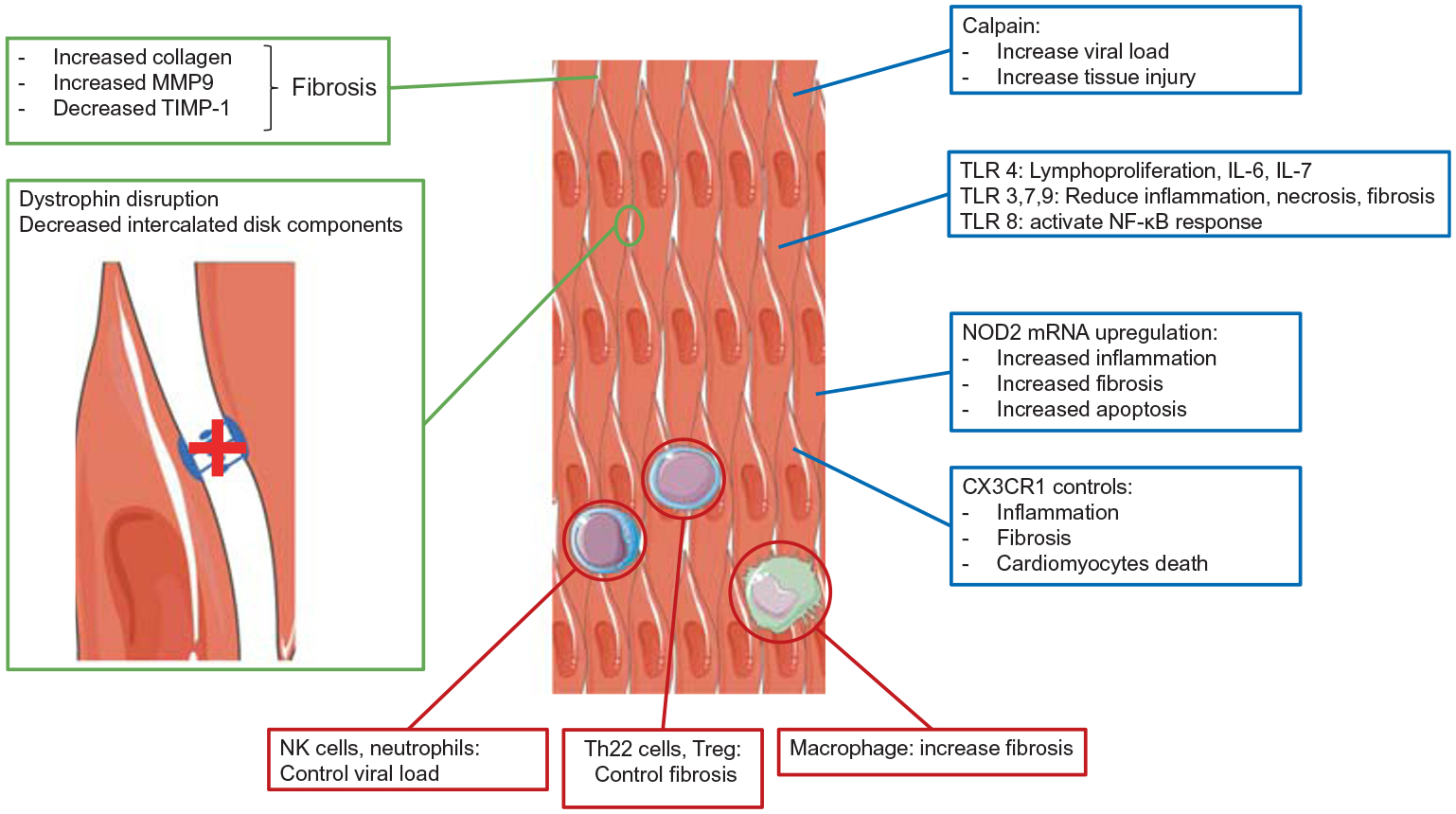
Remodeling of cardiac tissue by enteroviruses. Following infection and replication, cardiac tissues undergo significant changes. There are alterations in cell biology and the expression of many intracellular components, inducing a dysregulation of inflammation and immunity (shown as text in blue boxes). The immune system effectors will then be recruited and can act in an uncontrolled manner, triggering bystander effects (shown as text in red boxes). Ultimately, the extracellular space between cells will undergo drastic changes, impairing systolic function by breaking down the contacts and communication between cardiac cells (shown as text in green boxes)

Enterovirus are known to be responsible for cardiac diseases, more specifically group B enteroviruses [29, 30]. Following an acute infection, the virus can persist in the cardiac tissue and lead to chronic myocarditis and dilated cardiomyopathy. It is well documented that following enterovirus infection, cardiac tissues become inflamed and remodeling of the heart occurs [31]. More recently, extensive studies on the role of enteroviruses in persistent infections leading to myocarditis have been published (for example [32••],). A model that includes the more recent findings for cardiac tissue remodeling following enterovirus infections is displayed in Fig. 2. As illustrated in the figure, inflammation appears to play an important role in the pathogenesis of enterovirus-mediated heart disease. It has been reported that neutrophils recognize coxsackievirus B3 (CV-B3) and play a role in disease outcome. Although neutrophils can internalize CV-B3, the virus does not replicate well in these cells. Following entry, viruses are detected by endosomal TLR-8 and induce an NFκB response [33]. In a murine model, neutrophil depletion leads to reduced viral replication and cardiomyocyte hypertrophy [34]. Other immune cells are important for the remodeling of cardiac tissue. It has been shown that regulatory T cells (Treg) play a significant role in cardiac fibrosis. During infection, the Treg cell population decreases and fibrosis increases. Further studies with anti-IL-10 antibodies revealed that Treg modulation of fibrosis occurs via IL-10 secretion [35]. Additionally, Tsunoda and colleagues [36] found that TLR4-deficient mice had lower levels of lympho-proliferation, IL-6, and IL-17, all of which were associated with increased myocarditis susceptibility. Similarly, a mutation of Unc93b1, a chaperone protein for TLR3, TLR7, and TLR9, showed that lack of TLR signaling increased viral loads during CV-B3 infection and increased inflammation, necrosis, and fibrosis [37]. A murine model in animals lacking the chemokine receptor CX3CR1 also displayed increased inflammatory cytokines and chemokine expression, leading to a more extensive immune cell infiltrate, cardiac fibrosis, and cardiomyocyte death following CV-B3 infection [38]. Similarly, NKT cell-deficient mice exhibit a higher viral load and an increase in antiviral antibody titers during Theiler’s murine encephalomyelitis virus (TMEV) cardiac infection despite lower levels of lympho-proliferation and reduced IL-4 and IL-10 levels [39]. More recently, the role of IL-22-producing Th22 cells has been described during myocarditis. Following infection by CV-B3, mice expressed IL-22 at higher levels, as well as collagen type I-A1, collagen type III-A1, and matrix metalloproteinase-9, while levels of tissue inhibitor of metalloproteinase-1 (TIMP-1) were decreased. This resulted in cardiac fibrosis, a hallmark of chronic cardiac disease and tissue remodeling [40].

The mRNA levels for the cytoplasmic pattern recognition receptor NOD2 (nucleotide-binding oligomerization domain 2) have been shown to be upregulated in patients infected by CV-B3 and suffering from myocarditis. Interestingly, NOD2 knockdown decreased inflammatory infiltrate and pro-inflammatory cytokine production, fibrosis, apoptosis, and expression of the coxsackievirus and adenovirus receptor (CAR) [41]. Similarly, the calcium-binding alarmins S100A8 and S100A9, which function as damage-associated molecular patterns, were also upregulated in cardiac tissue of patients suffering from CV-B3-induced myocarditis. In vitro experiments showed that overexpression of these proteins enhanced oxidative stress and CV-B3 replication in cardiomyocytes and stimulated the expression of the chemokine, macrophage inflammatory protein-2 (MIP-2), in macrophages. Moreover, infection of mice deficient in S100A8 and S100A9 by CV-B3 showed improved left ventricular functions as well as a lower level of inflammation and viral replication [42].

Macrophages are known to play a major role in acute inflammation and chronic fibrosis. Mice depleted of macrophages were able to sustain increased levels of CV-B3 replication but had reduced pathology and lower levels of fibrosis. Interestingly, reduced levels of myocarditis and chronic fibrosis were also observed in mice knocked out for galectin 3, a lectin involved in pathogenic cardiovascular remodeling and autoimmune/inflammatory processes [43]; however, in this case viral titers were not increased [44].

The cellular cysteine protease calpain has been reported to act in enterovirus infections [45–47]. Inhibiting its action by using a transgenic mouse model overexpressing calpastatin led to a decrease in tissue injury and viral load. Moreover, the pro-inflammatory factors MPO, perforin, IFNγ, and IL-17 were downregulated as well as the fibrotic factors Smad3 and MMP2 [48]. This study also showed that calpain promotes fibroblast migration in vitro. Interestingly, calpains are also involved in CV-B-induced necrosis of polarized intestinal cells (Caco-2) by mediating tight junctions and actin cytoskeleton rearrangement [49].

Inflammation is the first response to cardiac infection, leading to remodeling of the tissue, but it also triggers changes in the cell itself. It is known that cardiac diseases are linked to changes in cell metabolism (reviewed in [50]), and it has been reported recently that following infection by CV-B3, infiltrating leukocytes activate NFκB signaling, inducing cytokine expression that turns down oxidative gene expression. This deficit impairs energy metabolism in cardiomyocytes as well as their functions [51]. More changes can be seen during myocardial infection of the heart; it is now well known that CV-B3 2A proteinase can disrupt dystrophin and prevent its membrane localization [52] and that expression of 2A alone is sufficient to trigger dilated cardiomyopathy [53]. More recently, it has been shown that the C-terminal fragment of dystrophin (a product of viral 2A proteolytic cleavage) is able to bind the sarcoglycan complex and prevent the natural function of dystrophin. A threshold of 50% of un-cleaved cardiac dystrophin is necessary to prevent cardiac disease [54]. Additionally, by inducing the expression of miR-21, the virus downregulates intercalated disk components, disrupting the connections and communications between cardiac cells, and more specifically by destabilizing desmosomes [55]. CV-B3 infection also promotes the production of collagen I/IV in neonatal rat cardiac fibroblasts, inducing cardiac fibrosis [56]. Interestingly, this effect could be reversed by activating adenosine monophosphate–activated protein kinase. However, it is unlikely that the reduction of fibrosis would be an effective treatment even though acute myocarditis is characterized by inflammation and fibrosis, which decrease during the chronic phase of myocarditis. This reduction does not correlate with a recovery of hemodynamic function in a murine model [57]. More importantly, it has been shown that CV-B3 can infect cardiac tissue from juvenile mice at a subclinical dose, without any detectable disease symptoms. But once they reach adulthood, the mice infected as juveniles are predisposed to experience heart hypertrophy, leading to heart failure. This might be explained by a depletion of cardiac progenitor cell pools following an early differentiation of cardiac progenitor cells induced by CV-B3 [58••].

## Hand, Foot, and Mouth Disease

Hand, foot, and mouth disease (HFMD) is characterized by sores in the mouth and blisters on the hands, feet, and legs. This disease is known to be caused by viral several enterovirus strains (e.g., EV-A71, EV-A68, CV-A6, CV-A16). The disease can be recapitulated in non-obese diabetic/severe combined immuno-deficient (NOD/SCID) mice, gamma interferon receptor (*ifngr*) knockout mice, and stat-1 knockout mice [59]. These infection models produced disease symptoms, including paralysis, and death rates that can be used for further studies. In addition to causing hand, foot, and mouth disease, enteroviruses like EV-A71 can infect the CNS. It has also been demonstrated that EV-A71 can infect and replicate in human microvascular endothelial cells and can be shed on both sides of these polarized cells. Moreover, actin cytoskeleton destruction, membrane remodeling, and cell death were observed along with an increase in permeability in a model of the blood-brain barrier [60]. Finally, another enterovirus (CV-A16) can upregulate the transcription of its receptor, scavenger receptor class B member 2 (SCARB2), in 293 T cells. This results in enhancement of subsequent rounds of infection, potentially facilitating co-infection and possible recombination with EV-A71 [61].

## Conclusions

In this review, we have summarized recent findings on inflammation and remodeling of airway and cardiac tissues during picornaviruses infections. Although most picornavirus infections appear to be asymptomatic or lead to mild disease syndromes such as the common cold, the outcomes of these infections can lead to long-lasting effects in susceptible individuals, potentially life threatening, with or without chronic infections. These long-lasting effects can happen months or even years after the initial infection without any symptoms observed before the final stages, making it hard to diagnose and treat in the early phases when the pathology is mild. As a result, the importance of enterovirus infections in chronic diseases has been underestimated for a long time and has only more recently been acknowledged in disease syndromes such as cardiomyopathy [30, 32••] and diabetes [62]. Nevertheless, due to the asymptomatic nature of some acute infections, it is unlikely that we can diagnose these pathologies in the early stages, as healthy populations will never get sampled. The therapeutic options are to develop preventive vaccination, as explained by Dunne et al. [63], or to find ways to reverse tissue damage and remodeling in patients. For both options, a comprehensive understanding of cell-specific replication determinants and the pathogenic effects on infected tissues is critical to better anticipate these events and design therapeutic strategies.
